# Lack of Thermogram Sharpness as Component of Thermographic Temperature Measurement Uncertainty Budget

**DOI:** 10.3390/s21124013

**Published:** 2021-06-10

**Authors:** Krzysztof Dziarski, Arkadiusz Hulewicz, Grzegorz Dombek

**Affiliations:** 1 Institute of Electric Power Engineering, Poznan University of Technology, Piotrowo 3A, 60-965 Poznan, Poland; grzegorz.dombek@put.poznan.pl; 2 Institute of Electrical Engineering and Industry Electronics, Poznan University of Technology, Piotrowo 3A, 60-965 Poznan, Poland; arkadiusz.hulewicz@put.poznan.pl

**Keywords:** thermography, measures of sharpness, thermographic camera

## Abstract

The number of components of a thermographic temperature measurement uncertainty budget and their ultimate contribution depend on the conditions in which the measurement is performed. The acquired data determine the accuracy with which the uncertainty component is estimated. Unfortunately, when some factors have to be taken into account, it is difficult to determine the value of the uncertainty component caused by the occurrence of this factor. In the case of a thermographic temperature measurement, such a factor is the lack of sharpness of the registered thermogram. This problem intensifies when an additional macro lens must be used. Therefore, it is decided to commence research to prepare an uncertainty budget of thermographic measurement with an additional macro lens based on the B method described in EA-4/02 (European Accreditation publications). As a result, the contribution of factors in the uncertainty budget of thermographic measurement with additional macro lens and the value of expanded uncertainty were obtained.

## 1. Introduction

When performing a thermographic temperature measurement, the indication of the thermal imaging camera is influenced by a number of factors [1]. Each of them should be considered when constructing an uncertainty budget for thermal imaging temperature measurement [2].

Measurement uncertainty is defined as a parameter (or parameters) characterizing how well the (essentially unique) true value of the measurand is believed to be known [3].

The most important factors that have an effect on the temperature read-out value, and that should be taken into account in the uncertainty budget are, emission factor value [4], reflected temperature [5], distance between the camera lens and the object under observation [6], ambient temperature [7], external optical system temperature [8], transmission of the external optical system [9,10], and relative humidity [11]. These are the factors whose contribution to the uncertainty budget can be easily determined from measurements or available literature.

Their effect on the temperature *ϑ_t_* read from thermogram has been widely described [12,13,14]. Furthermore, these values, if correctly determined, make it possible to minimize their effect on *ϑ_t_*. It is possible by selecting proper coefficients in the thermographic camera software [15]. Another factor to be taken into account in the uncertainty budget of the thermographic temperature measurement is the lack of sharpness of the registered thermogram [16].

The more unsharp the registered thermogram, the more the temperature read from the thermogram differs from the actual temperature [17]. Modern thermographic observation cameras are often equipped with automatic sharpness adjustment systems which work similarly to the systems used in digital cameras [18,19]. Therefore, the sharpness adjustment issue becomes particularly visible in the case of cameras which do not have such a system. These are cameras used for research purposes, e.g., in microscopic thermography [20,21,22].

**Figure 1:**
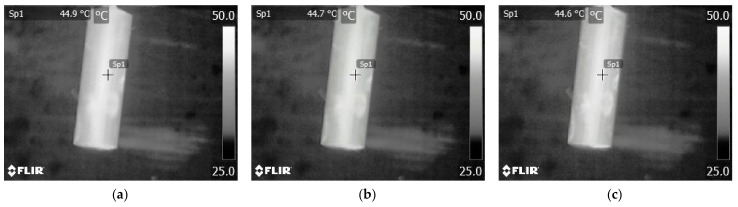
Thermograms presenting the Pt 1000 temperature sensor in a cylindrical case with a diameter of 3 mm. The thermograms are taken with the following distances between the lens and the object under observation: (**a**) 33 mm—distance between the additional thermographic camera lens and the observed object, as proposed by manufacturer. Temperature measured in Sp1 = 49.6 °C; (**b**) 33.1 mm. Temperature measured in Sp1 = 49.5 °C; (**c**) 34 mm—maximum allowable value of the distance between lens and object, which allows obtaining a sharp thermogram (by manufacturer). Temperature measured in Sp1 = 49.3 °C.

The sharpness adjustment issue becomes especially important in the case of thermographic temperature measurements of small objects of several millimeters [16]. Taking such measurements requires the use of an additional macro type wide-angle lens. Considering the small depth of focus of the macro lens, taking a thermographic temperature measurement of such small objects requires precision. This issue is illustrated by means of thermograms presented in Figure 1.

While observing the thermograms presented in Figure 1, it can be noticed that it is difficult to take a thermogram of a satisfactory quality. It is also easy to take a measurement, the value of which differs significantly from the actual value. It is not possible to minimize the effect of the out-of-focus thermogram on the temperature value read from the thermogram by an appropriate setting of the thermographic camera software [15]. An additional problem is that the amount of data to estimate the contribution of this factor to the uncertainty of the thermographic temperature measurement is insufficient. This problem is particularly evident when a measurement is taken with an additional macro lens.

There are well-known studies on the uncertainty of thermographic temperature measurement [23,24,25]. The B-type uncertainty method has also been applied for thermographic measurement temperature uncertainty determination [26,27]. The authors did not find any articles on the inclusion in the uncertainty budget of the factor related to the unsharpness of the registered thermogram. The necessary data were not available.

## 2. Materials and Methods

### 2.1. The Measurement System

The problem of insufficient data to estimate the contribution to the uncertainty budget of thermographic temperature measurement of a factor related to the unsharpness of the recorded thermogram was decided to be solved by carrying out research work. To carry out the measurements to determine the contribution of the out-of-focus factor in thermal imaging temperature measurements and the probability distribution for this factor, it was necessary to construct a measurement system.

In the course of the on-going measurements, the Flir E50 thermographic camera (Flir, Wilsonville, OR, USA) [28] equipped with an additional Close—up 2x up macro lens (Flir, Wilsonville, OR, USA) [29] was used. The measurement system had to be designed in such a way as to enable two options to change the sharpness of the registered thermogram: by changing the distance between the lens and the observed object *d* and by changing the focus adjustment ring angle placed on the thermographic camera lens *α*.

The ability to change the value *d* was achieved by attaching the camera to a bracket that was attached to the movable part of the linear guide. According to the information contained in the catalogue sheet provided by the manufacturer of the Close-up 2x lens, a sharp thermogram can be obtained when *d* = WD = 33 mm. WD (work distance) is the distance indicated by the manufacturer at which a sharp thermogram can be obtained and at which spatial resolution is known. The biggest permissible difference between WD and *d* could be ±0.4 mm. For this reason, the distance between the Close-up 2x lens and the object under observation was equal to 33 mm.

In order to achieve repeatable settings of the stepper motor which is part of the linear guide, it was controlled by a Siemens S1200 PLC controller (Siemens, Berlin, Germany). While changing the pre-set, the distance *d* was measured by means of the MMR30 [30] linear potentiometer. The sensor used allowed *d* to be measured with a satisfactory resolution of <0.01 mm.

Correct operation of the MMR30 required a low current to flow through this sensor. For this reason, a Howland current source was constructed. Source current value was *I_z_* = 100 µA. The voltage at the terminals of the MMR30 was measured by means of the appropriate components of the PLC controller.

The measured signal was converted into a digital signal through the ADC converter installed in the PLC controller. In order to use the full range of the ADC, the signal was amplified using an AD620 operating in a differential amplifier configuration. The system used, containing the AD620, allowed the gain of the operational amplifier *G* to be adjusted.

The value of *G* was selected so that the value of the largest measurable signal was at least 95% of the ADC range. Additionally, the system containing the ADC allowed for offset correction. Adjusting the input impedance was achieved by placing an additional operational amplifier working in the repeater circuit in the measurement path.

Measurement of a low voltage value at the MMR30 terminals meant that even a small amplitude interference induced on the test leads could affect the final measurement result. In order to avoid interference, filtration in the current source, filtration on the MMR 30 terminals by connecting a capacitor and an RC filter before the AD620 were used. An FTP shielded twisted pair cable was also used.

The possibility to change the angle *α* was achieved by using an additional stepper motor mounted on the arm placed on the movable part of the linear guide. The focus adjustment ring was coupled with the motor shaft via a rubber belt. The stepper motor was controlled by means of the PLC with a step of *α_k_* = 1.5°.

The observed element under observation was placed on a special-purpose table. The position angle *β* of the table plane was controlled by means of a stepper motor adjusted the PLC with a step of *β_k_* = 0.9°. The station so designed was placed in a chamber made of plexiglass. The external dimensions of the chamber were 45 cm × 35 cm × 35 cm, while the internal dimensions of the chamber were 40 cm × 30 cm × 30 cm. The chamber restricts air exchange with the environment. The walls of the chamber do not allow visible light to pass through. The chamber walls were lined with black foam made of polyurethane. The foam used is characterized by porous structure and every single pore of the foam resembles the black body cavity model.

**Figure 2:**
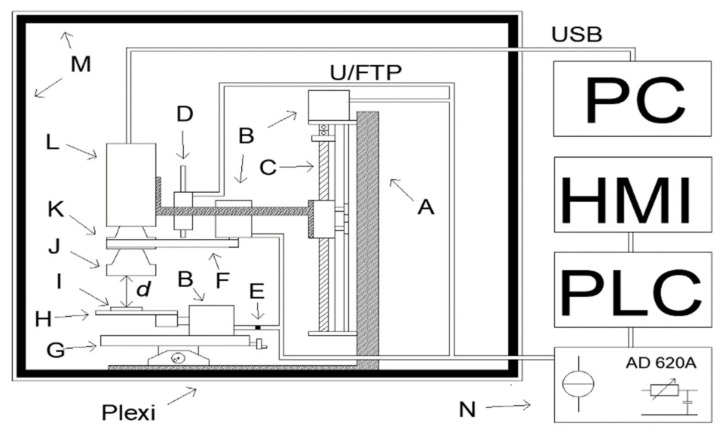
Schematic view of the measurement system: (A) tripod; (B) stepper motors; (C) linear guide; (D) resistance linear distance sensor; (E) connector; (F) rubber belt; (G) cross table; (H) additional table with adjustable angle position relative to thermal camera lens; (I) observed object; (J) additional macro lens: Close up lens 2x P/NT 197200; (K) thermal imaging camera lens; (L) thermal imaging camera; (M) polyurethane foam; (N) additional module with sources of current, filter systems and measurement amplifier; (d) WD (work distance)—distance between the observed object and thermal imaging camera macro lens = 33 mm.

The chamber walls so prepared are characterized by a high value of the emissivity factor *ε* = 0.95 [31] and a small value of the reflectance factor *ρ*. This causes a part of the measurement system closed in the chamber to be optically isolated. It additionally minimizes the reflection of the IR radiation from the plexiglass walls, the radiation originating from the measurement system elements, e.g., from stepper motors. The measurement system designed is presented in Figure 2.

The research performed included thermographic observations of an electronic element. It was a Pt1000 temperature sensor placed in a cylindrical case with a diameter of *ϕ* = 3 mm and length of *l* = 6 mm [32]. It was assumed that six series of thermograms would be taken—three series in which value *d* was changed within the range of WD 10 mm and three series in which value *α* was changed within the range of 0–45°.

The number of series was selected arbitrarily. All series were made one by one at the same time and under the same conditions. Thermograms were taken with an interval of 1 s. Due to the later comparison of thermograms sharpness by the observers, it was decided to make six series of thermograms. The analysis of a larger number of thermograms could be tedious for the observers—the survey would be too long and, consequently, it would be difficult to find people willing to fill it out.

The range of value *d* was selected arbitrarily as a range within which thermograms with different sharpness values were registered. At the same time, the selected range of *d* contained range *d* indicated by the manufacturer where a sharp thermogram (WD ± 0.4 mm) can be obtained. Range *α* contained all possible values for this thermographic camera. After each thermogram was taken, the temperature read from the thermogram was recorded.

**Figure 3:**
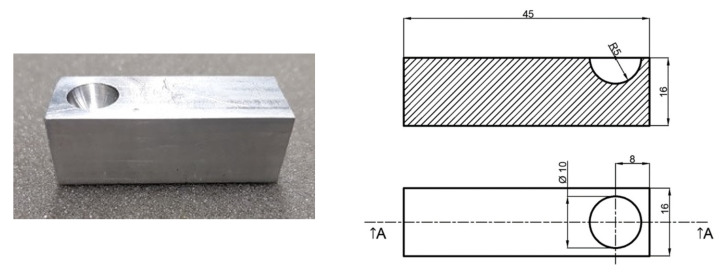
Photograph and dimensions of the reflector used to measure reflected radiation. The presented reflector is mounted in the place of the table with the observed element. Reflector built for the reflected radiation measurement. Dimensions are in millimeters.

The temperature of the observed sensor *ϑ_S_*, measured from the resistance of this sensor *R_s_* and the known function *ϑ_s_* = *f*(*R_s_*), was also recorded. Six series of thermograms were taken in total. Before taking all thermograms, the impact of radiation reflected from the observed element *IR_R_* had to be minimized. In this case, the main source of such radiation was the thermographic camera lens. In order to minimize the impact of *IR_R_*, a specially designed reflector was used. This was an aluminum block sized 16 mm × 16 mm × 45 mm with a hollowed-out semisphere with *R* = 5 mm. The photograph and the dimensions of the reflector built are presented in Figure 3.

In order to compensate for the reflected radiation, the table with the element under observation was replaced with the reflector presented in Figure 3. The semisphere was in the same plane in which the observed element was after the *IR_R_* impact had been minimized. After *d* = 0 and *ε* = 1 were entered into the camera software, the thermographic camera indication was read to be 32.7 °C. It was a measured value of the reflected temperature (reflected radiation) *ϑ_refl_* that was entered into the thermographic camera software. Then the reflector was replaced with the table with the element under observation. The value *ε* amounting to 0.42 was fixed based on the temperature read from the thermogram with temperature *ϑ_S_*.

Values of the air temperature inside the chamber *ϑ_a_* and air humidity *ω* were measured by means of a sensor installed inside the chamber. Values *ϑ_a_* and *ω* were 20 °C and 50% respectively. To minimize the impact of the other factors, *d* = 0 m, the external optical system temperature value *ϑ_l_* = *ϑ_a_* = 20 °C and the external optical system transmission value *τ_l_* equal to 1 were entered into the thermographic camera software. The so prepared thermographic camera was used to take all thermogram series.

### 2.2. Measures of Sharpness

In order to determine the effect of the lack of sharpness of the registered thermogram on the *ϑ_t_* value, the thermograms taken were analyzed by means of selected sharpness measures. It was decided to use simple measures of sharpness which employ the properties of the registered thermogram [33,34,35,36]. Measures that use transforms were not used [37,38,39].

Such decision was made to check whether simple measures of sharpness are suitable for the description of sharpness of thermograms presenting an element placed in a cylindrical enclosure. All measures of sharpness employed are presented by means of Equations (1)–(11). The first measure of sharpness to be used was the variance *D*^2^. This is the simplest of the sharpness measures used. For a thermogram sized *M* × *N*, the value *D*^2^ can be obtained by means of Equations (1) and (2) [33]:

(1) $$D^{2}=\frac{1}{M\times N}\overset{M-1}{\underset{x=0}{\sum }}\overset{N-1}{\underset{y=0}{\sum }}{\left(f\left(x,y\right)-\mu \right)}^{2}$$

where:

(2) $$-\mu =\frac{1}{M\times N}\overset{M-1}{\underset{x=0}{\sum }}\overset{N-1}{\underset{y=0}{\sum }}{\left(f\left(x,y\right)\right)}^{2}$$

Another measure of sharpness used called EOG (energy of gradient) used the first derivatives of the image in both vertical and horizontal directions. The EOG value was obtained by means of the following equation [33]:

(3) $$\mathrm{EOG}=\overset{M}{\underset{x}{\sum }}\overset{N}{\underset{y}{\sum }}{\left(f_{x}^{2}+f_{y}^{2}\right)}^{2}$$

Another measure used to describe sharpness of the registered thermogram was EOL (energy of Laplacian). It is a measure that employs the use of second derivatives in both directions: vertical and horizontal. The EOL value can be obtained by means of equation [33]:

(4) $$\mathrm{EOL}=\overset{M-1}{\underset{x=2}{\sum }}\overset{N-1}{\underset{y=2}{\sum }}{\left(f_{xx}^{2}+f_{yy}^{2}\right)}^{2}$$

SML (sum modified Laplacian) is another one of the sharpness measures that has been proposed in the literature sources. Nayar noticed that, in case of the Laplace operator, the second derivatives in the vertical and horizontal directions may have different signs. He suggested modified Laplacian (*ML*) of a discrete expression. *ML* can be expressed by means of Equation (5) [34]:

(5) $$\nabla_{ML}^{2}f\left(x,y\right)=\left|2f\left(x,y\right)-f\left(x-h,y\right)-f\left(x+h,y\right)\right|\;\;\;\;=\left|2f\left(x,y\right)-f\left(x-h,y\right)-f\left(x+h,y\right)\right|$$

In Equation (6), “*h*” means a step which always equaled 1 in the works performed. SML can be described by the following expression [33,34]:

(6) $$\mathrm{SML}=\overset{x+N}{\underset{i=x-N}{\sum }}\overset{y+N}{\underset{j=y-N}{\sum }}\nabla_{ML}^{2}f{\left(i,j\right)}^{2}\;\nabla_{ML}^{2}f{\left(i,j\right)}^{2}\geq T$$

where *N* defines the size of a window used to measure the sharpness of the thermogram.

The penultimate measure of sharpness is spatial frequency (*SF*). Spatial frequency is not a new measure of sharpness but a modified version of the sharpness measure using the energy of the gradient (EOG). *SF* can be defined by the following Equations (7)–(9) [33,36]:

(7) $$SF=\sqrt{{\left(RF\right)}^{2}+{\left(CF\right)}^{2}}$$

where *RF* (row frequency) is the row frequency [34]:

(8) $$RF=\sqrt{\frac{1}{M\times N}\overset{M}{\underset{x=1}{\sum }}\overset{N}{\underset{y=2}{\sum }}{\left[f\left(x,y\right)-f\left(x,y-1\right)\right]}^{2}}$$

where *CF* (column frequency) is respectively the column frequency [33]:

(9) $$CF=\sqrt{\frac{1}{M\times N}\overset{M}{\underset{x=2}{\sum }}\overset{N}{\underset{y=1}{\sum }}{\left[f\left(x,y\right)-f\left(x-1,y\right)\right]}^{2}}$$

The last measure to be used was Tenengrad. It is a measure using the Sobel operator to determine the gradient amplitude. In order to use this measure of sharpness, the following expression can be used [33]:

(10) $$Tenengrad=\overset{x=M-1}{\underset{x=2}{\sum }}\overset{x=N-1}{\underset{y=2}{\sum }}{\left(\nabla S\left(x,y\right)\right)}^{2},\nabla S\left(x,y\right)>T$$

where *T* is the discrimination threshold value, and ∇S(x,y) is the Sobel gradient value.

### 2.3. Methodology of Estimating Uncertainty by Type B Method

The B type uncertainty estimation method permits one to estimate the contribution of a specific factor to the uncertainty budget based on the measurements taken, experience, data available in the literature and in calibration certificates [40].

In this case, in order to estimate the uncertainty of temperature thermographic measurement factors influencing the value *ϑ_t_* should be defined. For this reason, the thermographic camera measurement equation was analyzed. The measurement equation one interrelates the output value and the input values. In the case of a thermographic camera, the output value is the total radiation reaching the camera lens *W_tot_*.

The input values are used to describe three components of the IR radiation reaching the camera lens: radiation emitted by the observed surface *W_obj_*, ambient radiation reflected from the observed surface *W_refl_*, and radiation of the atmosphere surrounding the observed surface *W_a_* [41]:

(11) $$W_{tot}=\varepsilon \times \tau_{a}\times W_{obj}+\left(1-\varepsilon \right)\times \tau_{a}\times W_{refl}+\left(1-\tau_{a}\right)\times W_{a}$$

where *τ_a_* is atmosphere transmittance.

An additional Close up-2x lens was used for the work performed. Therefore the radiation emitted by this lens *W_l_* should be taken into account. For this purpose, it is required to know the temperature of the lens *ϑ_l_* and the lens transmittance *τ_l_.* When the additional lens is taken into account, Equation (11) will take the form of:

(12) $$W_{tot}=\varepsilon \times \tau_{a}\times W_{obj}\times \tau_{l}+\left(1-\varepsilon \right)\times \tau_{a}\times W_{refl}\times \tau_{l}+\left(1-\tau_{a}\right)\times W_{a}\times \tau_{l}+\left(1-\tau_{l}\right)\times W_{l}$$

**Figure 4:**
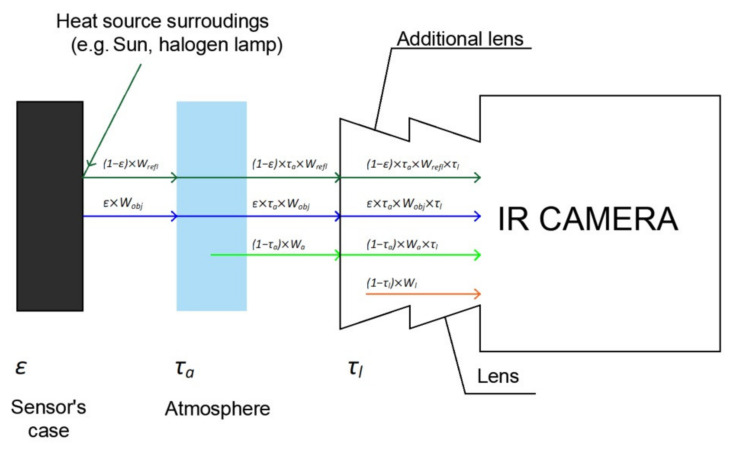
Components of the total IR radiation reaching the lens: radiation reflected from the observed object (dark green), IR radiation emitted by the observed object (blue), IR radiation emitted by the atmosphere between the thermographic camera and the observed object (bright green), IR radiation emitted by the additional macro lens.

All components of the IR radiation reaching the camera lens are presented in Figure 4.

When the Stefan–Boltzmann law is complied, Equation (12) takes the form of:

(13) $$W_{tot}=\varepsilon \times \tau_{a}\times \sigma \times \vartheta_{obj}^{4}\times \tau_{l}+\left(1-\varepsilon \right)\times \tau_{a}\times \sigma \times \vartheta_{refl}^{4}\times \tau_{l}+\left(1-\tau_{a}\right)\times \sigma \times \vartheta_{a}^{4}\times \tau_{l}+\left(1-\tau_{l}\right)\times \sigma \times \vartheta_{l}^{4}$$

where *σ* is the Boltzmann constant equal to 5.67 cm × 10^−8^ W/(m^2^·K^4^) [41].

Finally, after carrying out the transformations, the equation which makes it possible to calculate the temperature based on the total radiation reaching the camera lens can be obtained [41]:

(14) $$\vartheta_{obj}=\sqrt[{4}]{\frac{W_{tot}-\left(1-\varepsilon \right)\times \tau_{a}\times \sigma \times \vartheta_{refl}^{4}\times \tau_{l}-\left(1-\tau_{a}\right)\times \sigma \cdot \vartheta_{a}^{4}\times \tau_{l}-\left(1-\tau_{l}\right)\times \sigma \times \vartheta_{l}^{4}}{\varepsilon \times \tau_{a}\times \sigma \times \tau_{l}}}$$

(15) $$\tau_{a}\left(d,\omega \right)=K_{a}\times \exp\left[-\sqrt{d}\times \left(\alpha_{1}+\beta_{1}\sqrt{\omega }\right)\right]+\left(1-K_{a}\right)\times \exp\left[-\sqrt{d}\times \left(\alpha_{2}+\beta_{2}\sqrt{\omega }\right)\right]$$

(16) $$\omega (\omega_{\%},\vartheta_{a})=\omega_{\%}\times \exp\left(h_{1}+h_{2}\times \vartheta_{a}+h_{3}\times \vartheta_{a}^{2}+h_{4}\times \vartheta_{a}^{3}\right)$$

where *ω* is the coefficient indicating the amount of water vapor in the atmosphere, *ω*% is relative humidity, *K_atm_* = 1.9 is atmosphere damping factor, *α*_1_ and *α*_2_ are damping factors for an atmosphere without water vapor, *β*_1_ and *β*_2_ are damping factors for water vapor *h*_1_ = 1.5587, *h*_2_ = 6.939 × 10^−2^, *h*_3_ = −2.7816 × 10^−4^, and *h*_4_ = 6.8455 × 10^−7^.

After analyzing Equations (14)–(16), the value range should be determined for each quantity in these equations. After determining their value ranges, it is possible to derive the estimate input quantity using Equation (17):

(17) $$x_{i}=\frac{1}{2}\left(a_{+}+a_{-}\right)$$

where *a*_+_ is the upper range limit, *a*_−_ is the lower range limit, *x_i_* is an estimate obtained. [42] Input quantity should be understood as the quantities to the right of Equations (14)–(16). Then, after calculating the estimate of input quantity, the standard uncertainty related to the considered input quantity should be calculated.

The standard uncertainty value is the positive variance root as defined by the following equation:

(18) $$u^{2}(x_{i})=\frac{1}{12}{\left(a_{+}+a_{-}\right)}^{2}$$

If the difference between the values is 2*a*, Equation (18) takes the form of Equation (19) [42].

(19) $$u^{2}(x_{i})=\frac{1}{3}{\left(a\right)}^{2}$$

Then a probability distribution must be specified for each quantity. Probability distribution is a function giving the probability that a random variable takes any given value or belongs to a given set of values [42].

Then it is possible determine the uncertainty contribution that is related to the analyzed input quantity appearing in the equation. It is equal to the standard uncertainty associated with input quantity and sensitivity coefficient c.

Coefficient *c* describes the effect of the changes in the value of input quantity estimate on the value of output quantity estimate. Coefficient *c* can be calculated as a constituent derivative of the measurement function in relation to the input quantity [42].

There is also another way to determine the coefficient *c* by means of numerical methods. For this purpose, one should calculate changes of the output quantity estimate caused by a change in the estimate *x_i_* of the input quantity by +*u*(*x_i_*) and −*u*(*x_i_*). The obtained difference in the output quantity estimate *y* should be divided by 2*u*(*x_i_*). Contribution of uncertainty of the input quantity *u_i_*(*y*) = *u*(*x_i_*)×*c* [42].

The standard uncertainty associated with output quantity is the square root of the uncertainty contributions. Output quantity should be understood as the values on the left side of Equations (14)–(16). This value can be obtained from the following equation

(20) $$u^{2}\left(y\right)=\overset{N}{\underset{i=1}{\sum }}u_{i}^{2}\left(y\right)$$

where: *u*(*y*)—standard uncertainty connected with output quantity [43].

Expanded uncertainty *U*(*y*) is the product of the standard uncertainty associated with the output quantity and the coverage factor.

Coverage factor is a number larger than one by which a combined standard measurement uncertainty is multiplied to obtain an expanded measurement uncertainty [42].

(21) $$U\left(y\right)=u\left(y\right)$$

The value of *τ_a_* in Equation (14) can be obtained from Equation (15), while the value of *ω* in Equation (15) can be obtained from Equation (16). For this reason, when estimating the uncertainty of a thermovision temperature measurement, it is first necessary to estimate the standard uncertainty value for *ω* (*u*(*ω*)) taking into account the quantities in Equation (16).

Then the value of *u*(*ω*) should be taken into account when estimating the value of standard uncertainty *τ_a_* (*u*(*τ_a_*)). The value of *u*(*τ_a_*) should be estimated taking into account the quantities in Equation (15).

Finally, this value should be taken into account when estimating the expanded uncertainty of the thermal imaging temperature *U*($\vartheta_{obj}$). The value of *U*($\vartheta_{obj}$) should be estimated taking into account the quantities from Equation (14). The constructed budget is presented later in the article.

## 3. Experimental Results

### 3.1. Comparison of Sharpness Measurement Results and Observer Indications

The values of the thermograms’ measures of sharpness determined by means of Equations (1)–(10) have been standardized. This enabled the obtained results to be credibly compared. For this purpose, the relations in Equation (22) were used.

(22) $$V^{\prime }=\frac{V-V_{min}}{V_{max}-V_{min}}$$

where *V*′ is obtained standardized value, *V_min_* is lowest values in the specific series, and *V_max_* is highest value in the specific series.

**Figure 5:**
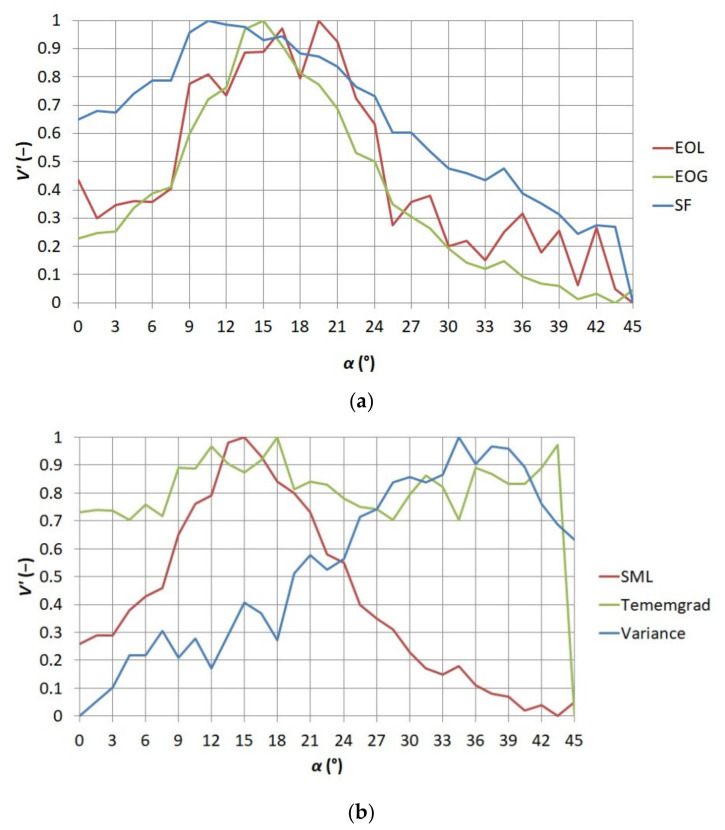
Relationship between the standardized value of sharpness measures *V*′ and the angle of focusing ring mounted on the thermographic camera lens *α* for the first series of thermograms (**a**) EOL, EOG, and SF (**b**) SML, Tenengrad, and Variance.

**Figure 6:**
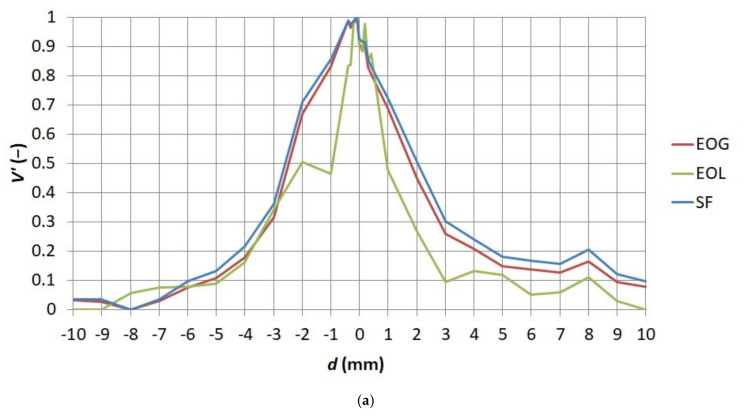

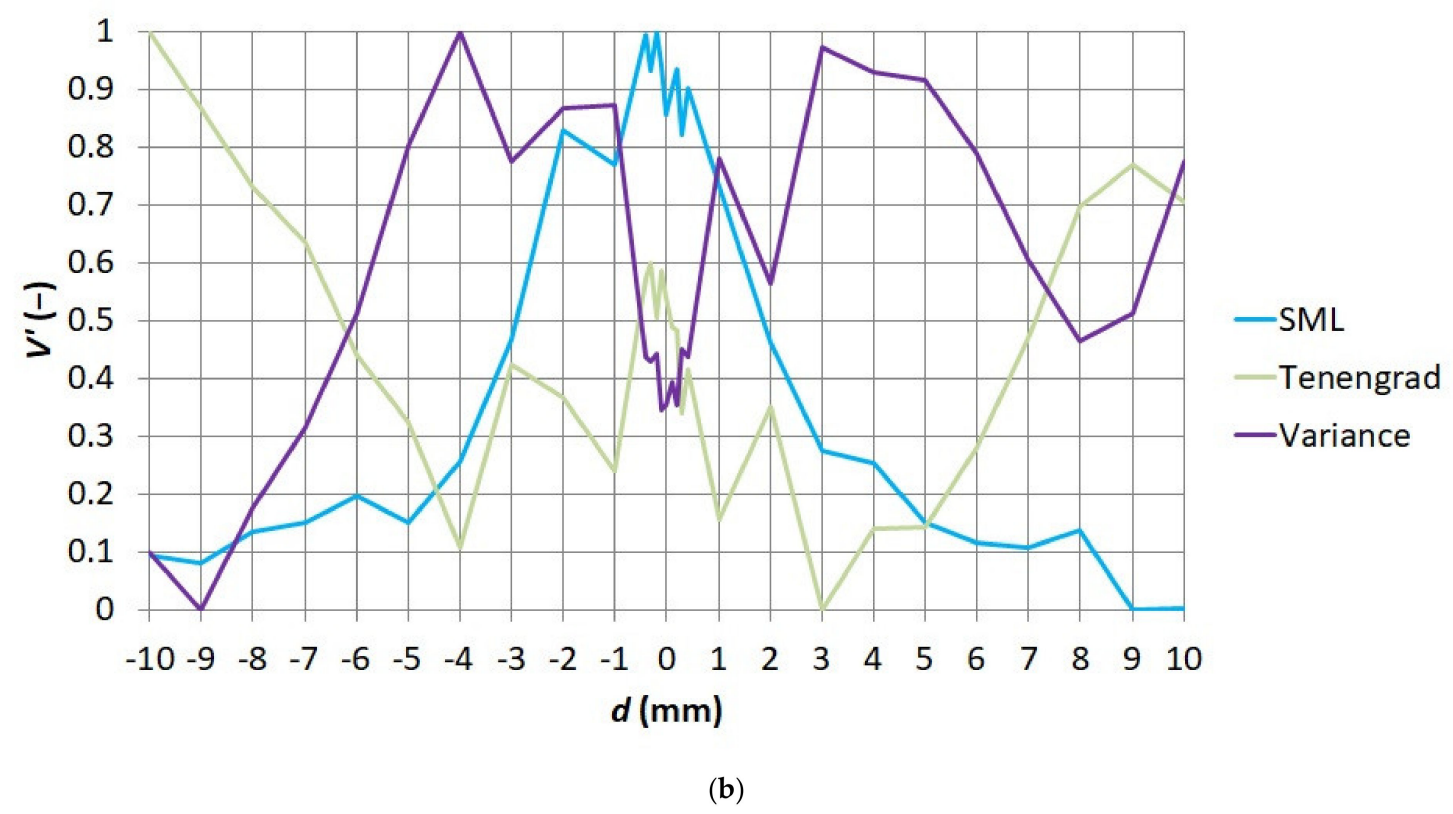
Relationship between the standardized value of sharpness measures *V*′ and the distance between the thermographic camera lens and observed object *d* for the first series of thermograms (**a**) EOL, EOG, and SF (**b**) SML, Tenengrad, and Variance.

Figure 5 and Figure 6 present the standardized values of the obtained measures of sharpness. It was decided to present results obtained for the first series to show the distribution of the standardized values of each of the measures in function *V*′ *= f*(*α*) and to present results obtained for the fourth series to show the distribution of standardized values of each of the measures in function *V*′ *= f*(*d*).

In order to assess the correct modelling of thermogram sharpness by means of sharpness measures (1–10), thermograms in all series were presented to a group of 137 volunteers of both sexes aged from 20 to 24. Each of the volunteers took part in the research on a voluntary basis. Each observer’s task was to indicate the sharpest thermogram. Completed questionnaires were collected over a period of several months.

**Figure 7:**
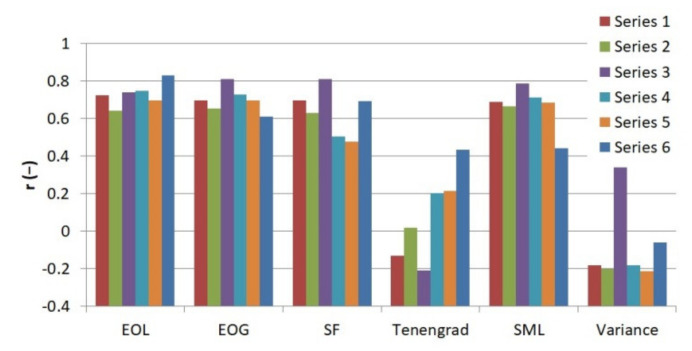
Comparison of coefficients of correlation between the values of sharpness measures (functions V′ = f(α) and V′ = f(*d*)) and the observers’ indications for every series of thermograms and each measure of sharpness, where V’—standardized value of sharpness measures, *α*—angle of focusing ring placed on the thermographic camera lens, *d*—distance between the thermographic camera lens and observed object.

Then the standardized values of the measures of sharpness of all thermograms were compared to the observers’ indications. For this purpose, coefficients of correlation between the standardized values of sharpness measures of each thermogram (functions *V*′ = *f*(*α*) and *V*′ = *f*(*d*)) and observers’ indications were calculated. The obtained values of coefficients of correlation are presented in Figure 7.

**Figure 8:**
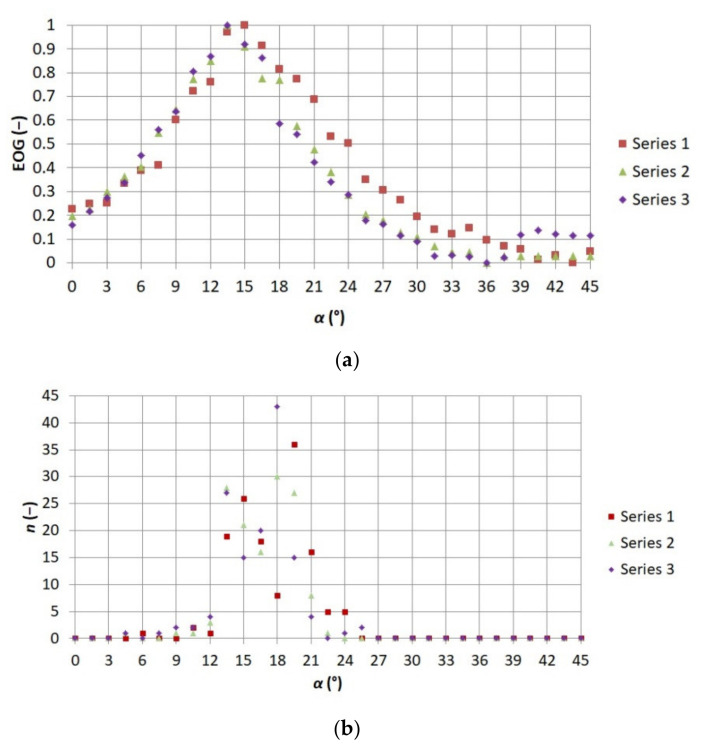
Comparison of relationship (**a**) between EOG measure of sharpness and the angle of focus adjustment ring placed on the thermographic camera lens *α* 1–3 and (**b**) between numbers of thermograms indicated by observers as sharp *n* and adjustment ring angle on the thermographic camera lens *α*.

**Figure 9:**
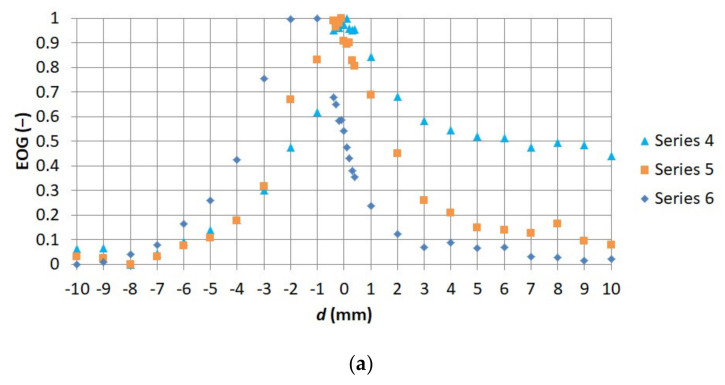

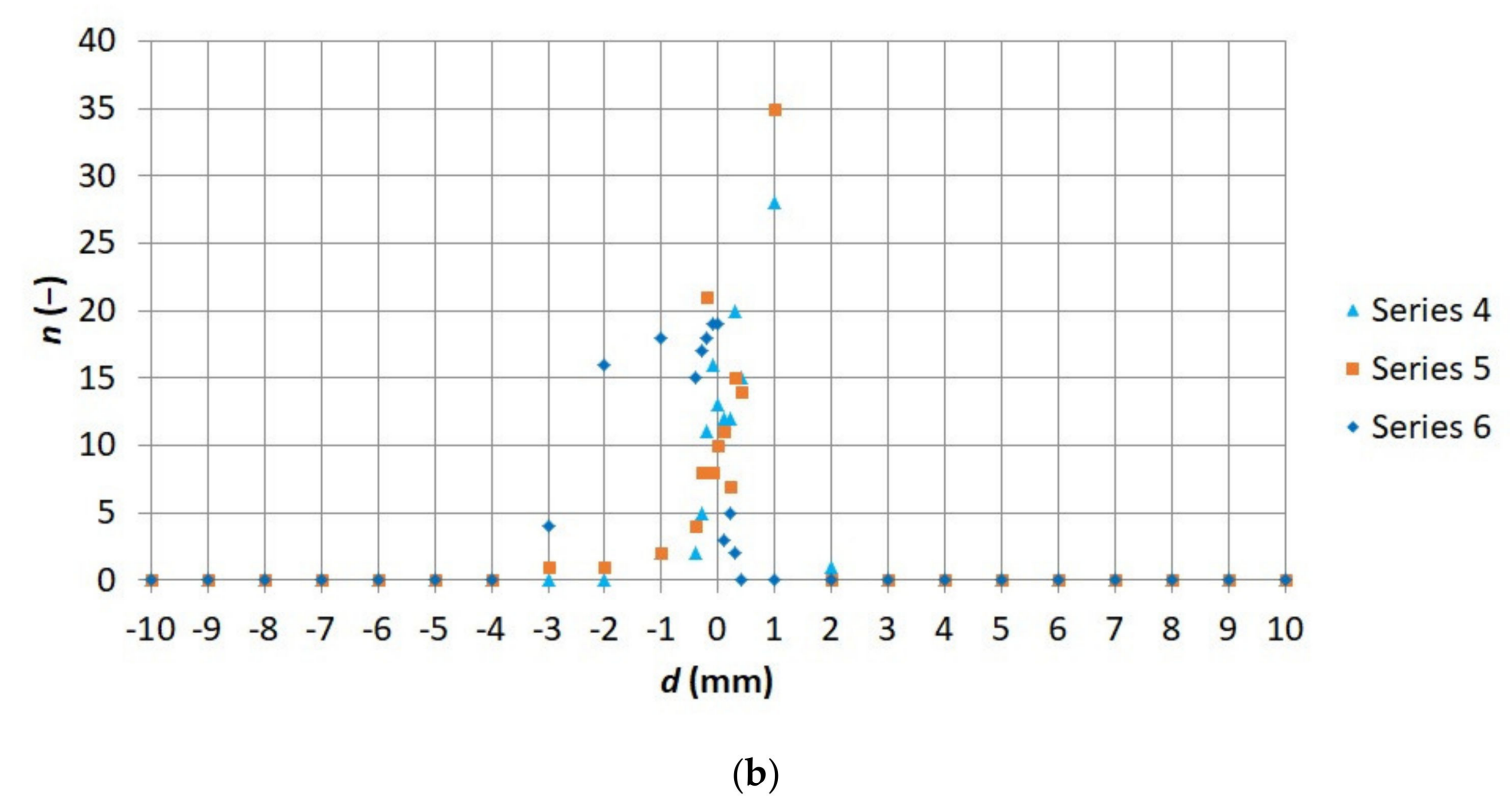
Comparison of relationship (**a**) between EOG measure of sharpness and distance between 1–3 (**b**) between numbers of thermograms indicated by observers as sharp *n* and distance between the thermographic camera lens and observed object.

Consequently, it was possible to select such a measure of sharpness which corresponded best to observer indications. It was noticed that the highest values of coefficients of correlation between the observers’ indications and the values of the measures of sharpness were obtained by means of EOG and EOL. EOG was selected for further works, with the level of difficulty of using individual measures of sharpness being considered. Figure 8 and Figure 9 present a comparison of the standardized EOG values for each of the series of thermograms with observer indications. The number of thermograms indicated by observers as sharp is marked as n.

**Figure 10:**
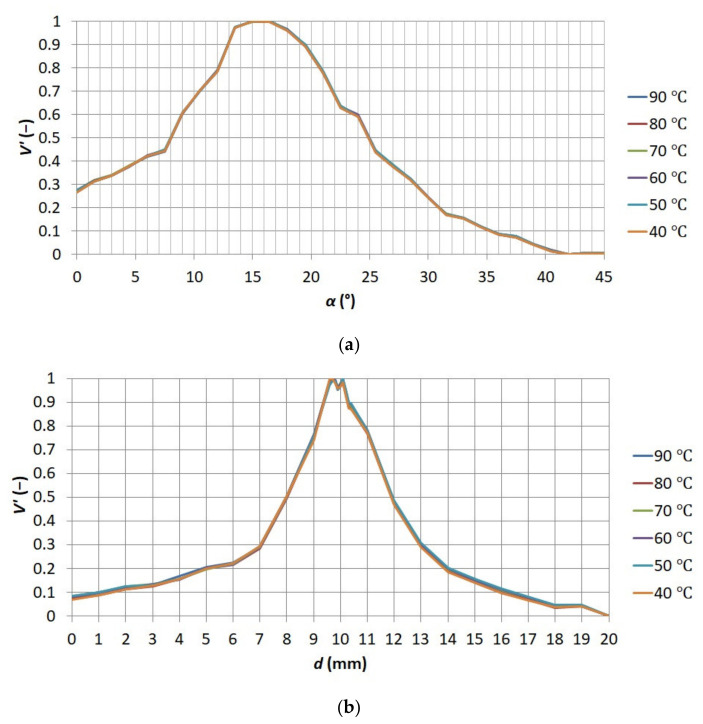
Relationships (**a**) between standardized sharpness measure *V′* and adjustment ring angle *α* placed on the thermographic camera lens for various temperatures of the sensor (**b**) between standardized sharpness measure *V’* and distance *d* between thermographic camera lens and observed object for various temperatures of the sensor.

Then it was verified that the distribution of functions *V*′ = *f*(*α*) and *V*′ = *f*(*d*) did not change together with the temperature of the sensor under observation. The distribution of both functions for various values of the sensor temperature is presented in Figure 10.

**Figure 11:**
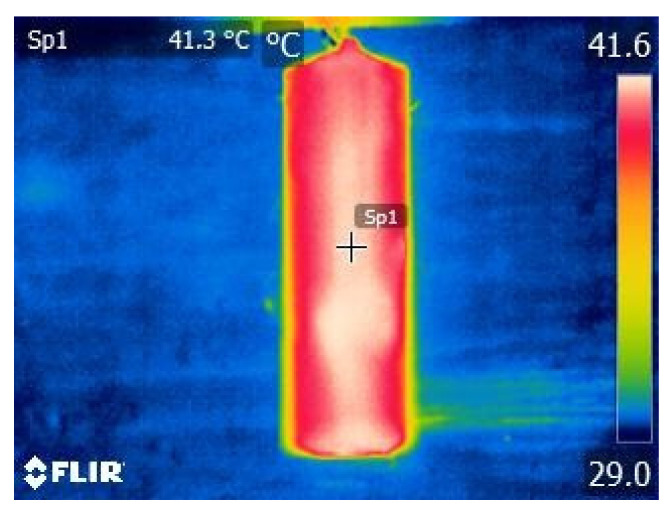
Placement of the measurement point SP_1_ on each thermogram while the measurements are in progress.

As a next step, temperature *ϑ_S_* was compared to *ϑ_t_*. Value *ϑ_S_* was assumed to be the correct value. Value *ϑ_S_* was read before each measurement. In this way, the possibility of changes in the value of *ϑ_S_* in the course of the measurements was considered. Figure 11 shows a point on the thermogram from which the *ϑ_t_* value was read.

Based on the measured values of *ϑ_S_* and *ϑ_t_* for every thermogram, the absolute error value of the thermographic temperature Δ*ϑ* was calculated according to Formula (23).

(23) $$\Delta \vartheta =\Delta \vartheta_{t}-\Delta \vartheta_{s}$$

**Figure 12:**
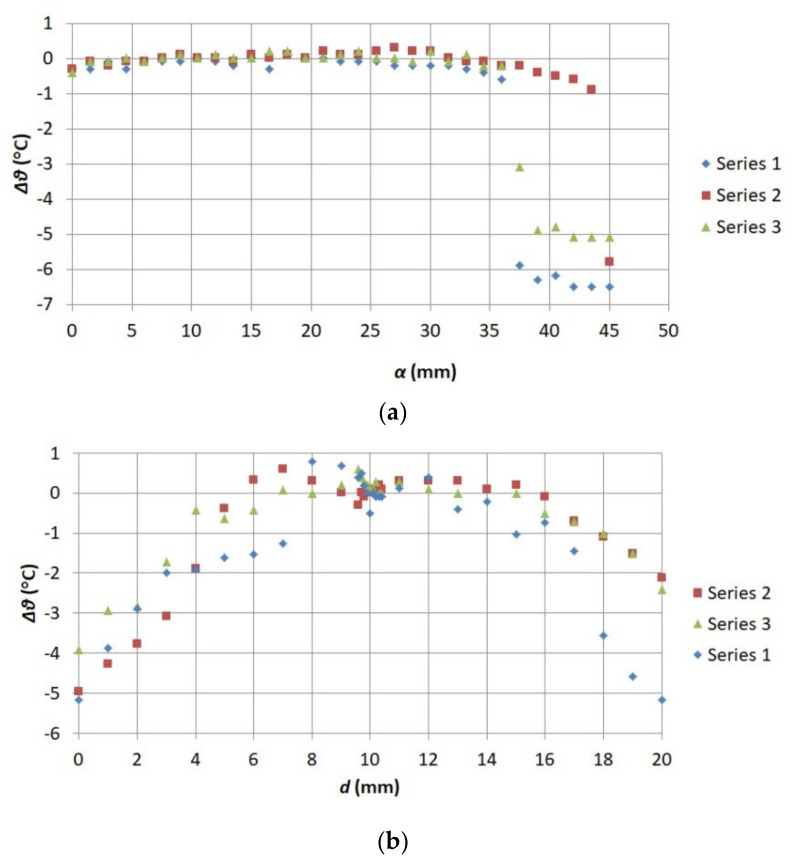
Relationship between the absolute error value of the thermographic temperature and (**a**) focus adjustment ring angle on the thermographic camera lens *α*, (**b**) distance between the lens and the observed object *d*.

Calculated values Δ*ϑ* in function *d* and in function *α* are presented in Figure 12.

### 3.2. The Uncertainty Budget

The results presented in Figure 8, Figure 9 and Figure 12 made it possible to check how much the thermographic temperature measurement uncertainty determined without taking into account the thermogram sharpness *u*(*ϑ_t_*) differs from the thermographic temperature measurement uncertainty determined with the thermogram sharpness *u*(*ϑ_t_*)*_s_* being taken into account.

First, an uncertainty budget that did not take into account the thermogram unsharpness was constructed. The uncertainty budget was evaluated based on the principles shown in 2.3 and [42]. At the beginning, the range of variability *ω* was checked. It was decided that the range of variability *ω* would be determined for extreme conditions observed in the laboratory, i.e., for temperatures within a range of 18–35 °C and for humidity within a range of 30–60%.

Using Equation (16), simulation tests were carried out. It was found that *ω* took the lowest value amounting to 4.56 for *ϑ_a_* = 18 °C and *ω*% = 30% and the highest value amounting to 23.29 for *ϑ_a_* = 35 °C and *ω*% = 60%. Knowing the upper and lower range *ω* made it possible to determine the estimate *ω* according to Equation (17).

Equation (16) consists of two variables and constants. Therefore the designed uncertainty budget contains two quantities—*ϑ_a_* and *ω*%. Estimate *ϑ_a_*, similarly to estimate *ω*%, was determined by means of Equation (17). In order to determine variances *ϑ_a_* being squares of standard uncertainties, Equation (18) was used. In order to determine variances *ω*% being squares of standard uncertainties, Equation (19) was used.

Knowing the upper and lower range and the identical probability of occurrence for all values *ϑ_a_* and *ω*% made it possible to match a rectangular distribution of probability for these properties.

Sensitivity factor obtained by numeric method is described in point 2.3 and in [42]. The obtained values of coefficients *c* and contributions of uncertainty *u*(*ω*) are presented in Table 1. Evaluation of uncertainty budget in tables can be seen in other articles [44,45,46].

The value of *u*(*ω*) shown in the bottom right corner of the table was determined by means of Formula (22).

Based on the Central Limit Theorem, values *ω* were attributed to the normal distribution of probability. Then, the process of determining value *u*(*τ_a_*) was commenced. It should be noted that it is only possible to obtain the value *τ_a_* by means of Equation (15) after the value *ω* is calculated based on Equation (16). Therefore the uncertainty budget designed for *τ_a_* is superior to the budget designed for *ω*.

Values from Table 1 will be used to design it. After analyzing Equation (15), one may notice that, apart from the constants, it also contains the variable *d*. The estimate *d* and *τ_a_* was determined by means of (17) while the variance *d* was determined by means of (18). Knowing the limits of the range *d* and assuming the same probability for all values to occur, a rectangular distribution of probability was assumed. The value *τ_a_* was attributed to the normal distribution based on the Central Limit Theorem. Value (*τ_a_*) was obtained by means of (20). The obtained values *c* and contributions of uncertainty *u*(*τ_a_*) are presented in Table 2.

After the uncertainty *τ_a_* (bottom right corner in Table 2) had been determined, it was possible to design the major uncertainty budget presented in Table 3. Therefore it was necessary to define the limits of the range of variables which occurred in Equation (14). Information on the limits of variable ranges came from the experiments performed and from literature sources. Value *ε* was changed within a range of 0.95–0.98, value *ϑ_refl_* within a range from 25 °C to 35 °C. The assumed value *τ_l_* was the value of transmittance of the materials used for the construction of thermographic cameras working within the LWIR (long wave infrared) band—0.9–1.

The additional lens was installed on the thermographic camera. Therefore, the assumption of *ϑ_l_* = *ϑ_a_* was made. Consequently, the values of *ϑ_l_* were considered within a range from 18 °C to 35 °C. The last value range was determined for *W_tot_*. This value was determined by means of (14). Values of the variables in Equation (14) were changed within pre-determined ranges. In consequence, the highest value *W_tot_* = 0.1669 and the lowest value *W_tot_* = 0.1439 were determined. Coefficient *c* which is the constituent derivative of Equation (14) is presented in Equations (24)–(30).

(24) $$\frac{\partial \vartheta_{obj}}{\partial \tau_{a}}=\frac{\frac{-\tau_{l}\vartheta_{a}^{4}\sigma -\tau_{l}\vartheta_{refl}^{4}\sigma \left(1-\varepsilon \right)}{\tau_{l}\sigma \tau_{a}\varepsilon }-\frac{-\tau_{l}\vartheta_{refl}^{4}\sigma \tau_{a}\left(1-\varepsilon \right)-\tau_{l}\vartheta_{a}^{4}\sigma \left(1-\tau_{a}\right)-\left(1-\tau_{l}\right)\vartheta_{l}^{4}\sigma +W_{tot}}{\tau_{l}\sigma \tau_{a}^{2}\varepsilon }}{4{\left(\frac{-\tau_{l}\vartheta_{refl}^{4}\sigma \tau_{a}\left(1-\varepsilon \right)-\tau_{l}\vartheta_{a}^{4}\sigma \left(1-\tau_{a}\right)-\left(1-\tau_{l}\right)\sigma \vartheta_{l}^{4}+W_{tot}}{\tau_{l}\sigma \tau_{a}\varepsilon }\right)}^{\frac{3}{4}}}$$

(25) $$\frac{\partial \vartheta_{obj}}{\partial \varepsilon }=\frac{\frac{\vartheta_{refl}^{4}}{\varepsilon }-\frac{-\tau_{l}\vartheta_{refl}^{4}\sigma \tau_{a}\left(1-\varepsilon \right)-\tau_{l}\vartheta_{a}^{4}\sigma \left(1-\tau_{a}\right)-\left(1-\tau_{l}\right)\vartheta_{l}^{4}\sigma +W_{tot}}{\tau_{l}\sigma \tau_{a}\varepsilon^{2}}}{4{\left(\frac{-\tau_{l}\vartheta_{refl}^{4}\sigma \tau_{a}\left(1-\varepsilon \right)-\tau_{l}\vartheta_{a}^{4}\sigma \left(1-\tau_{a}\right)-\left(1-\tau_{l}\right)\sigma \vartheta_{l}^{4}+W_{tot}}{\tau_{l}\sigma \tau_{a}\varepsilon }\right)}^{\frac{3}{4}}}$$

(26) $$\frac{\partial \vartheta_{obj}}{\partial W_{tot}}=\frac{1}{4\tau_{l}\sigma \tau_{a}\varepsilon {\left(\frac{-\tau_{l}\vartheta_{refl}^{4}\sigma \tau_{a}\left(1-\varepsilon \right)-\tau_{l}\vartheta_{a}^{4}\sigma \left(1-\tau_{a}\right)-\left(1-\tau_{l}\right)\vartheta_{l}^{4}\sigma +W_{tot}}{\tau_{l}\sigma \tau_{a}\varepsilon }\right)}^{\frac{3}{4}}}$$

(27) $$\frac{\partial \vartheta_{obj}}{\partial \vartheta_{refl}}=\frac{\vartheta_{refl}^{3}\left(1-\varepsilon \right)}{\varepsilon {\left(\frac{-\tau_{l}\vartheta_{a}^{4}\sigma \left(1-\tau_{a}\right)-\left(1-\tau_{l}\right)\vartheta_{l}^{4}\sigma -\tau_{l}\vartheta_{refl}^{4}\sigma \tau_{a}\left(1-\varepsilon \right)+W_{tot}}{\tau_{l}\sigma \tau_{a}\varepsilon }\right)}^{\frac{3}{4}}}$$

(28) $$\frac{\partial \vartheta_{obj}}{\partial \vartheta_{l}}=-\frac{\vartheta_{l}^{3}\left(1-\tau_{l}\right)}{\sigma \tau_{a}\varepsilon {\left(\frac{-\tau_{l}\vartheta_{a}^{4}\sigma \left(1-\tau_{a}\right)-\left(1-\tau_{l}\right)\sigma \vartheta_{l}^{4}-\tau_{l}\vartheta_{refl}^{4}\sigma \tau_{a}\left(1-\varepsilon \right)+W_{tot}}{\tau_{l}\sigma \tau_{a}\varepsilon }\right)}^{\frac{3}{4}}}$$

(29) $$\frac{\partial \vartheta_{obj}}{\partial \vartheta_{a}}=-\frac{\vartheta_{a}^{3}\left(1-\tau_{l}\right)}{\tau_{a}\varepsilon {\left(\frac{-\tau_{l}\vartheta_{a}^{4}\sigma \left(1-\tau_{a}\right)-\left(1-\tau_{l}\right)\sigma \vartheta_{l}^{3}-\tau_{l}\vartheta_{refl}^{4}\sigma \tau_{a}\left(1-\varepsilon \right)+W_{tot}}{\tau_{l}\sigma \tau_{a}\varepsilon }\right)}^{\frac{3}{4}}}$$

(30) $$\frac{\partial \vartheta_{obj}}{\partial \tau_{l}}=\frac{\frac{\vartheta_{refl}^{4}\sigma \tau_{a}\left(-\left(1-\varepsilon \right)\right)-\vartheta_{a}^{4}\sigma \left(1-\tau_{a}\right)+\sigma \vartheta_{l}^{4}}{\tau_{l}\sigma \tau_{a}\varepsilon }-\frac{-\tau_{l}\vartheta_{refl}^{4}\sigma \tau_{a}\left(1-\varepsilon \right)-\tau_{l}\vartheta_{a}^{4}\sigma \left(1-\tau_{a}\right)-\left(1-\tau_{l}\right)\vartheta_{l}^{4}\sigma +W_{tot}}{\tau_{l}^{2}\sigma \tau_{a}\varepsilon }}{4{\left(\frac{-\tau_{l}\vartheta_{refl}^{4}\sigma \tau_{a}\left(1-\varepsilon \right)-\tau_{l}\vartheta_{a}^{4}\sigma \left(1-\tau_{a}\right)-(1-\tau_{a})\vartheta_{l}^{4}\sigma +W_{tot}}{\tau_{l}\sigma \tau_{a}\varepsilon }\right)}^{\frac{3}{4}}}$$

The standard uncertainty *u*(*ϑ_obj_*) was determined by means of (20) and it is provided in the bottom right corner of Table 3. The expanded uncertainty *U*(*ϑ_obj_*) was obtained by multiplying the standard uncertainty by the coverage factor *k* = 2. Value *U*(*ϑ_obj_*) was 1.11 °C.

### 3.3. Uncertainty Budget with Thermogram Sharpness

In order to check how much the value *U*(*ϑ_obj_*) will change after taking into account the lack of sharpness of the registered thermogram, one more component was added to the uncertainty budget presented in Table 3, representing the value shown by the thermographic camera depending on the lack of sharpness of the registered thermogram *ϑ_us_*.

The value *ϑ_us_* was determined based on the analysis of results presented in Figure 11. In addition, this value was added to Equation (14) as a correction. After the correction was added, Equation (14) took the form of Equation (31). The highest value Δ*ϑ* was assumed to be the correction value applied in the uncertainty budget. It was decided that the range of *ϑ_us_* variability would be considered separately for every series and each method used to change the lack of thermogram sharpness.

When unsharpness changed as a result of change in *d*, the value Δ*ϑ_max_* = *ϑ_us_* equaled consecutively: 3.9 °C in the first series, 4.9 °C in the second series, and 5.25 °C in the third series. In the case of unsharpness changes due to changes in *α*, the range *ϑ_us_* equaled consecutively: −5.10 °C in the fourth series, −5.9 °C in the fifth series, −6.5 °C in the sixth series. Based on the results of the experiments performed, it can be concluded that a normal distribution of probability can be attributed to quantity *ϑ_us_*. Values of the estimate *ϑ_us_* and the standard uncertainty *u*(*ϑ_us_*) were obtained by means of Equations (17) and (20). The maximum range obtained from experiment (Figure 11) for each series was substituted as the upper range limit while 0 was substituted as the lower limit. The value *c* for the uncertainty component related to *ϑ_us_* was calculated numerically in accordance with the principles described in point 2.3 and in [42]. Moreover, in this case the value *u*(*ϑ_us_*) was calculated by means of (18). The thermographic camera lens position angle against the object under observation was not changed during the course of the works. Therefore the factor related to this angle was not considered.

(31) $$\vartheta_{obj}=\sqrt[{4}]{\frac{W_{tot}-\left(1-\varepsilon \right)\;\;\tau_{a\;}\times \sigma \times \vartheta_{refl}^{4}\times \tau_{l}-\left(1-\tau_{a}\right)\;\times \;\sigma \times \vartheta_{a}^{4}\times \tau_{l}-\left(1-\tau_{l}\right)\times \sigma \times \vartheta_{l}^{4}}{\varepsilon \times \tau_{a}\times \sigma \times \tau_{l}}}+\vartheta_{us}$$

An exemplary uncertainty budget for Pt1000 placed in a cylindrical case when the lack of sharpness of the registered thermogram was changed as a result of the change in *α* is presented in Table 4.

After taking *ϑ_us_* into account, the value *U*(*ϑ_us_*) for *k* = 2 is 6.59. Table 5 presents the values *U*(*ϑ_us_*) obtained for both sensors and both ways to change the lack of sharpness of the registered thermogram.

## 4. Conclusions

There are several factors that have an effect on the standard uncertainty of thermographic temperature measurement. While analyzing values of standard uncertainties and sensitivity coefficients of particular components of the uncertainty budget presented in Table 5, one may notice that the factor having a significant contribution to the determined uncertainty is the lack of sharpness of the registered thermogram.

Factors that also largely contribute to the standard uncertainty of thermographic temperature measurement include the reflected temperature and transmittance of material of which the additional lens is made. Transmittance of material of which the additional lens is made can be equated with the transmittance of the transmission window.

This proves that while carrying out the thermographic temperature measurement of an electronic element using an additional Close up 2x lens, special care must be taken to correctly set the sharpness of the registered thermogram and correctly compensate for the reflected temperature. This is particularly important because the sharpness of a registered thermogram cannot be corrected after it has been taken.

Some of the factors that should be taken into account while producing such an uncertainty budget of thermographic temperature measurement with an additional lens are of marginal importance. These include ambient temperature and humidity. The change in the temperature value read from the thermogram caused by the lack of thermogram sharpness differs depending on the cause of such unsharpness.

It is worth noting that the thermogram is not sharp when the distance between the lens and the observed object has been incorrectly selected and the position angle of the focus adjustment ring is incorrectly adjusted. Consequently, the lack of sharpness of the thermogram has a major effect on the temperature value read from the thermogram. In such a case, the contribution of the factor related to the changes in the camera indication resulting from increased unsharpness of the registered thermogram will be significant.

The contribution of this factor will decrease as the sharpness of the registered thermogram improves. This shows that in order to perform such a thermographic temperature measurement of an electronic element, which suffers from the smallest possible error, one should correctly adjust the sharpness of the registered thermogram.

In this article results were obtained by Type B evaluation of uncertainty described in EA-4/02 (European Accreditation publications). In future studies these results should be compared with the results obtained with the use of other methods, for example, Monte Carlo.

## Figures and Tables

**Table 1:** Uncertainty budget *ω*.

Symbol *X_i_*	Unit	Estimate of Quantity *x_i_*	Standard Uncertainty *u*(*x_i_*)	Distribution of Probability	Sensitivity Coefficient *c_i_*	Contribution of Uncertainty *u_i_*(*y*)
*ϑ_a_*	°C	26.50	4.90	rectangular	0.62	3.04
*ω*%	%	44.50	17.32	rectangular	0.25	4.33
*ω*	-	13.93				5.29

**Table 2:** Uncertainty budget *τ_a_*.

Symbol *X_i_*	Unit	Estimate of Quantity *x_i_*	Standard Uncertainty *u*(*x_i_*)	Distribution of Probability	Sensitivity Coefficient *c_i_*	Contribution of Uncertainty *u_i_*(*y*)
*ω*	-	13.93	5.29	normal	−3.78 × 10^−5^	−0.003
*d*	m	0.033	0.0057	rectangular	−0.0204	−0.0007
*τ_a_*	-	0.9987				0.0010

**Table 3:** Major uncertainty budget determined for the quantity *ϑ_obj_*. Change in the lack of sharpness through the change of *α* (series 3).

Symbol *X_i_*	Unit	Estimate of Quantity *x_i_*	Standard Uncertainty *u*(*x_i_*)	Distribution of Probability	Sensitivity Coefficient *c_i_*	Contribution of Uncertainty *u_i_*(*y*)
*τ_a_*	-	0.9987	0.0010	normal	0.4488	0.0004
*W_tot_*	W/m^2^	0.1554	0.0066	rectangular	67.7701	0.4472
*ε*	-	0.97	0.0086	rectangular	−7.6450	−0.0657
*ϑ_refl_*	°C	30	2.8868	rectangular	0.0119	0.0344
*τ_l_*	m	0.95	0.0289	rectangular	−10.3189	0.2982
*ϑ_a_*	°C	26.5	4.9000	rectangular	−0.0151	−0.0740
*ϑ_l_*	°C	26.5	4.9000	rectangular	−0.0151	−0.0740
*ϑ_obj_*	°C	41.3574				0.5525

**Table 4:** Major uncertainty budget determined for the quantity *ϑ_obj_* considering *ϑ_us_* for Pt1000 in a cylindrical case. Change of unsharpness by the change of *α* for series 3. The standard uncertainty value is in the bottom right corner.

Symbol *X_i_*	Unit	Estimate of Quantity *x_i_*	Standard Uncertainty *u*(*x_i_*)	Distribution of Probability	Sensitivity Coefficient *c_i_*	Contribution of Uncertainty *u_i_*(*y*)
*τ_a_*	-	0.9987	0.0010	normal	0.4488	0.0004
*W_tot_*	W/m^2^	0.1554	0.0066	rectangular	67.7701	0.4472
*ε*	-	0.97	0.0086	rectangular	−7.6450	−0.0657
*ϑ_refl_*	°C	30	2.8868	rectangular	0.0119	0.0344
*τ_l_*	m	0.95	0.0289	rectangular	−10.3189	0.2982
*ϑ_a_*	°C	26.5	4.9000	rectangular	−0.0151	−0.0740
*ϑ_l_*	°C	26.5	4.9000	rectangular	−0.0151	−0.0740
*ϑ_us_*	°C	3.25	1.88	normal	1	1.63
*ϑ_obj_*	°C	41.3574				3.25

**Table 5:** Obtained values *U*(*ϑ_us_*) for *k* = 2.

Method Used to Change the Lack of Sharpness	Series	*U*(*ϑ_us_*)
by the change of *d*	1	1.95
by the change of *d*	2	5.02
by the change of *d*	3	5.37
by the change of *α*	4	5.21
by the change of *α*	5	6.00
by the change of *α*	6	6.59

## Data Availability

Not applicable.
